# Spontaneous evolution of a massive hematoma caused by type 1 neurofibromatosis: Case report

**DOI:** 10.1016/j.ijscr.2020.01.016

**Published:** 2020-01-23

**Authors:** H. Ardhaoui, S. Halily, M. Mahtar

**Affiliations:** Department of Oto-Rhino-Laryngology, Head and Neck Surgery, Casablanca University Hospital, Casablanca, Morocco

**Keywords:** Neurofibromatosis, Hematoma, Internal jugular vein

## Abstract

**Introduction:**

Type1 Neurofibromatosis (NF1) is an autosomal dominant disorder with a great variety of clinical features. Vascular manifestations appear in less than 7%, and venous complications are extremely rare.

**Case presentation:**

We report a case of an enormous cervical hematoma caused by bleeding from the internal jugular vein in a NF1 patient. As the patient refused surgery, natural evolution was marked by spontaneous fistulization and drainage of the mass.

**Discussion:**

Several cases of fragility of both the vessel wall and the surrounding tissue are discussed and two jugular vein aneurysms were reported. Management of such cases remains unclear and some reports describe defective hemostatic control at surgery.

**Conclusion:**

Surgery in NF1 vascular involvement should be considered on a case by case basis especially when the risk of iatrogenesis is high. Surgeons need to be aware of hemorrhagic risks, which could occur because of vessel friability.

## Highlights

• Vascular complications are the second cause of death in type 1 Neurofibromatosis.
• Spontaneous fistulzation of the hematoma avoided the patient surgical morbidity.
• Venous system involvement is extremely rare in type 1 Neurofibromatosis and surgery should be considered on a case by case basis especially when the risk of iatrogenesis is high.

## 1 Introduction

Type1 Neurofibromatosis (NF1) is an autosomal dominant disorder caused by a mutation in chromosome 17 in the 17q11 region. It represents 95% of all neurofibromatosis with a prevalence of 1/4500 without sex distinction. Oto-laryngologic involvement occurs in 1–10% of cases [1,2]. Vascular manifestations appear in less than 7%, and venous complications are extremely rare [3,4].

We report a case of an enormous cervical hematoma caused by bleeding from the internal jugular vein and its spontaneous evolution. The work has been reported in line with the SCARE criteria [15].

## 2 Case report

**Fig. 1:**
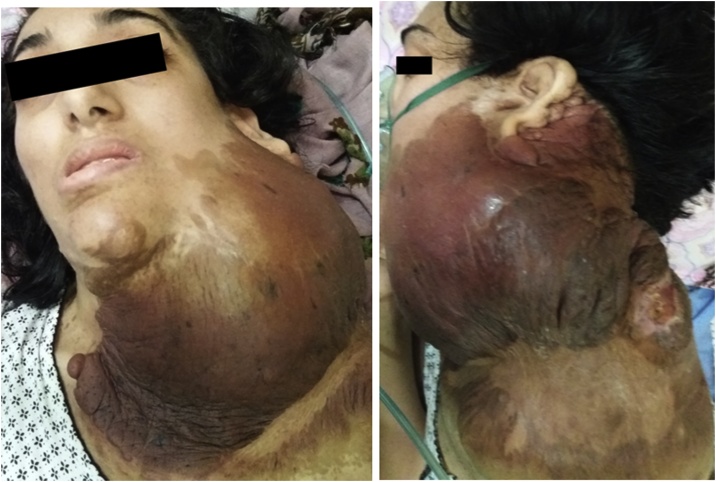
Enormous left cervical mass in NF1 patient with saggy hyper pigmented skin.

We present a case of a 27 years old woman with a NF1 who presented to the emergency room for a left cervical mass evolving for 48 h and rapidly increasing in size. The condition caused her breathing difficulties and dysphagia due to limited mouth opening. Physical examination revealed a 20 cm soft, non-pulsatile mass of the neck extending anteriorly from the pinna to the left supra-clavicular region and shoulder and posteriorly to the back of the neck. There was no thrill or bruit. The skin over the mass was hyper pigmented and saggy in favor of plexiforme neurofibromas (Fig. 1). Puncture brought a hematic liquid.

Doppler ultrasound revealed a large heterogeneous hypoechoic mass with a slightly increased vascular flow without a clear visualization of the carotid artery and internal jugular vein.

**Fig. 2:**
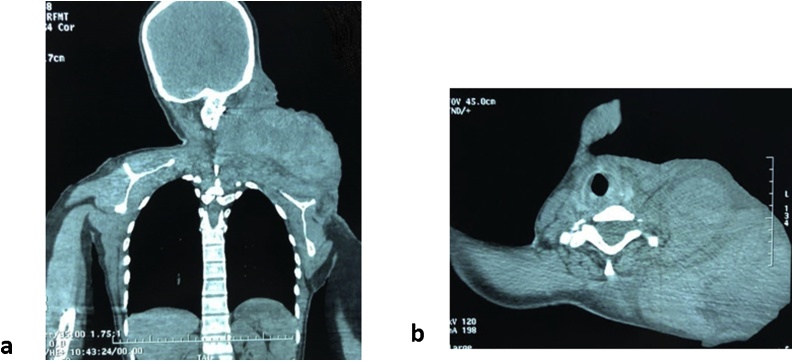
**a:** CT scan coronal section showing distal extension of the mass. **b:** Axial CT scan of the neck showing the mass with a permeable airway.

CT angiography showed a large mass of liquid density extending from the peri-auricular region to the left clavicle and shoulder compressing the oropharynx but the rest of the airway remained permeable. No arterial involvement was detected. The hematoma extended along the length of the internal jugular vein which was thrombosed. It measures approximately 22 × 16 × 11 cm (Fig. 2).

Surgery was indicated in consultation with cardiovascular surgeons. However, the patient refused surgery when the risks of uncontrolled bleeding were explained.

Evolution was marked by a spontaneous fistulization of the mass after two weeks and spontaneous progressive drainage of the hematoma thus disappearance of the compressive signs without necessity of transfusion. The patient did not have any recurrence or local complication at her one year follow up.

## 3 Discussion

Neurofibromatosis represents a heterogeneous group of seven diseases sharing the same cutaneous signs due to a common embryological origin [5].

Neurofibromatosis Type 1 (NF1), or von Recklinghausen disease, is an autosomal dominant disorder affecting one in 4500 individuals. NF1 affect mainly connective and nerve tissues yet, any organ can be involved [6]. Even though vascular involvement ranges between 0.4%–6.4% in NF1, vascular complications are the second cause of death, after malignant peripheral nerve sheath tumor [[3], [4], [5], [6], [7]].

On a review of the literature, several cases of fragility of both the vessel wall and the surrounding tissue are discussed [8,9] and two jugular vein aneurysms were also discussed [10,11]. Oderich et al. showed a predominant arterial involvement, in renal artery alone 41%, neck and head vessels (19%) and abdominal aorta (12%) [4].

Yilmaz et al. confirmed by histology the vein wall infiltration by NF1 which was mainly responsible for difficulty in hemostasis thus led to severe intraoperative bleeding [12]. Vascular invasion was the main cause of fatal or near fatal hemorrhages [13]. Despite that the NF1 infiltrates vessels, malignancy, cellular pleomorphism, or mitoses were not found in histological examination [14].

Regarding management, some authors reported surgical resection of venous aneurysms in NF1 patients [16]. However, other reports describe excessive vascular fragility and defective hemostatic control at surgery [17,18].

This case study describes a patient with NF1 who developed a venous bleeding causing a neck mass without aneurysm or arterial involvement. The natural evolution was marked by fistulization gradual decrease in mass’s size and without complications. This probably treated her condition better than surgery would. It avoided a serious threat of massive hemorrhage as major vessel thrombosis would enhance morbidity and mortality.

## 4 Conclusion

Surgery in NF1 vascular involvement should be considered on a case by case basis especially when the risk of iatrogenesis is higher. Surgeons need to be aware of haemorrhagic complications, which could occur because of vessel friability. More importantly, the patient should be informed of the risks and involved in therapeutic decisions.

## Sources of funding

None.

## Ethical approval

The study is exempt from ethical approval in our institution as it is a “Case report” and not a research study.

## Consent

Written informed consent was obtained from the patient for publication of this case report and accompanying images. A copy of the written consent is available for review by the Editor-in-Chief of this journal on request.

## Author contribution

**H. Ardhaoui**: Investigation, Resources, Writing - original draft, Writing - Review & Editing, Visualization.

**S. Halily**: Investigation, Resources, Writing - Review & Editing.

**M. Mahtar**: Validation, Supervision.

## Registration of research studies

This is a Case report that does not require a research registry.

## Guarantor

H. Ardhaoui, S. Halily.

## Provenance and peer review

Not commissioned, externally peer-reviewed.

## Declaration of Competing Interest

None.

## References

[1] Heuze Y., Piot B., Mercier J. (2002). Difficultés de la prise en charge chirurgicale des manifestations faciales de la neurofibromatose de type 1 ou maladie de von Recklinghausen chez l’enfant. Rev. Stomatol. Chir. Maxillofac..

[2] Wolkenstein P., Decq P. (1998). Les neurofibromatoses. Neurochirurgie.

[3] Hamilton S.J., Friedman J.M. (2000). Insights into the pathogenesis of neurofibromatosis 1 vasculopathy. Clin. Genet..

[4] Oderich G.S., Sullivan T.M., Bower T.C., Gloviczki P., Miller D.V., Babovic-Vuksanovic D. (2007). Vascular abnormalities in patients with neurofibromatosis syndrome type 1: clinical spectrum, management, and results. J. Vasc. Surg..

[5] Riccardi V.M. (1992). Neurofibromatosis: Phenotype, Natural History, and Pathogenesis.

[6] National Institutes of Health Consensus Development Conference (1988). Neurofibromatosis. Arch. Neurol..

[7] Rasmussen S.A., Yang Q., Friedman J.M. (2001). Mortality in neurofibromatosis 1: an analysis using U.S death certificates. Am. J. Hum. Genet..

[8] Hopsu E., Tarkkanen J., Vento S.I. (2009). Acquired jugular vein aneurysm. Int. J. Otolaryngol..

[9] Hinsch N., Kriener S., Ritter R.G. (2008). Fatal haemorrhage due to extensive fragility of medium- and large- sized arteries and veins in a young patient with neurofibromatosis 1. Cardiovasc. Pathol..

[10] Belcastro M., Palleschi A., Trovato R.A., Landini R. (2011). A rare case of internal jugular vein aneurysmal degeneration in a type 1 neurofibromatosis complicated by potentially life-threatening thrombosis. J. Vasc. Surg..

[11] Nopajaroonsri C., Lurie A.A. (1996). Venous aneurysm, arterial dysplasia, and near-fatal hemorrhages in neurofibromatosis type I. Hum. Pathol..

[12] Yilmaz M., Ada E., Yayvada H., Barutcu A. (1997). Management of a large arteriovenous fistula in the face: a case of neurofibromatosis type I. Ann. Plast. Surg..

[13] Larrieu A.J., Hashimoto S.A., Allen P. (1982). Spontaneous massive haemothorax in von Recklinghausen’s disease. Thorax.

[14] Hiraki T., Higashi M., Goto Y. (2014). A rare case of internal jugular vein aneurysm with massive hemorrhage in neurofibromatosis type1. Cardiovasc. Pathol..

[15] Agha R.A., Borrelli M.R., Farwana R., Koshy K., Fowler A., Orgill D.P., For the SCARE Group (2018). The SCARE 2018 statement: updating consensus Surgical CAse REport (SCARE) guidelines. Int. J. Surg..

[16] Delvecchio K., Moghul F., Patel B., Seman S. (2017). Surgical resection of rare internal jugular vein aneurysm in neurofibromatosis type 1. World J. Clin. Cases.

[17] Schievink W.I., Piepgras D.G. (1991). Cervical vertebral artery aneurysms and arteriovenous fistulae in neurofibromatosis type 1: case reports. Neurosurgery.

[18] Takahashi K., Maruyama A., Aina S. (1989). A case of ruptured left subclavian artery associated with von Rechlinghausen’s disease. Kyobu Geka.

